# Energy Scaling Advantages of Resistive Memory Crossbar Based Computation and Its Application to Sparse Coding

**DOI:** 10.3389/fnins.2015.00484

**Published:** 2016-01-06

**Authors:** Sapan Agarwal, Tu-Thach Quach, Ojas Parekh, Alexander H. Hsia, Erik P. DeBenedictis, Conrad D. James, Matthew J. Marinella, James B. Aimone

**Affiliations:** ^1^Microsystems Science and Technology, Sandia National LaboratoriesAlbuquerque, NM, USA; ^2^Sensor Exploitation, Sandia National LaboratoriesAlbuquerque, NM, USA; ^3^Center for Computing Research, Sandia National LaboratoriesAlbuquerque, NM, USA

**Keywords:** resistive memory, memristor, sparse coding, energy, neuromorphic computing

## Abstract

The exponential increase in data over the last decade presents a significant challenge to analytics efforts that seek to process and interpret such data for various applications. Neural-inspired computing approaches are being developed in order to leverage the computational properties of the analog, low-power data processing observed in biological systems. Analog resistive memory crossbars can perform a parallel read or a vector-matrix multiplication as well as a parallel write or a rank-1 update with high computational efficiency. For an *N* × *N* crossbar, these two kernels can be *O*(*N*) more energy efficient than a conventional digital memory-based architecture. If the read operation is noise limited, the energy to read a column can be independent of the crossbar size (*O*(1)). These two kernels form the basis of many neuromorphic algorithms such as image, text, and speech recognition. For instance, these kernels can be applied to a neural sparse coding algorithm to give an *O*(*N*) reduction in energy for the entire algorithm when run with finite precision. Sparse coding is a rich problem with a host of applications including computer vision, object tracking, and more generally unsupervised learning.

## Introduction

As transistors start to approach fundamental physical limits and Moore's law slows down, new devices and architectures are needed to enable continued computing performance gains (Theis and Solomon, 2010). The computational ability of current microprocessors is limited by the power they consume. For data intensive applications, the computational energy is dominated by moving data between the processor, SRAM (static random access memory), and DRAM (dynamic random access memory). New approaches based on memristor or resistive memory (Chua, 1971; Waser and Aono, 2007; Strukov et al., 2008; Kim et al., 2012) crossbars can enable the processing of large amounts of data by significantly reducing data movement. One of the most promising applications for resistive memory crossbars is brain-inspired or neuromorphic computing (Jo et al., 2010; Ting et al., 2013; Hasan and Taha, 2014; Chen et al., 2015; Kim et al., 2015). The brain is perhaps the most energy-efficient computational system known, requiring only 1–100 femtoJoules per synaptic event (Merkle, 1989; Laughlin et al., 1998), efficiently solving complex problems such as pattern recognition on which conventional computers struggle. Consequently, there has been great interest in making neuromorphic hardware (Cruz-Albrecht et al., 2013; Merolla et al., 2014). Resistive memories can effectively model some properties of neural synapses and the crossbar structure allows for high-density interconnectivity as found in the brain. For example, individual neurons in the cerebral cortex can receive roughly 10,000 input synapses from other neurons (Schüz and Palm, 1989).

Resistive memories are essentially programmable two terminal resistors. If a higher write voltage is applied to the device, the resistance will increase or decrease based on the sign of the voltage, allowing the resistance to be programmed. Consequently, it can be used to model a synapse. Its resistance acts like a weight that modulates the voltage applied to it. This has resulted in a large interest in developing neuromorphic systems based on it (Jo et al., 2010; Ting et al., 2013; Hasan and Taha, 2014; Kim et al., 2015). Each cell also has a very small area and the memory can be stacked in 3d when arranged in a crossbar structure. Therefore, industry is developing resistive memories to use as a digital replacement for flash memory (Jo et al., 2009; Chen, 2013; Chen et al., 2014; Cong et al., 2015).

A pressing question is whether neural-inspired computing systems are able to offer any resource advantage over more conventional digital computing systems. Neural-inspired systems are likely to take the form of a massively parallel collection of neuromorphic computing elements or cores that are each much simpler than conventional CPUs (Merolla et al., 2014). Conventionally, each neuromorphic core is based on a local SRAM memory array. This allows for data to be locally stored where it is used, eliminating the need to move large amounts of data. Simply organizing the computing system in this manner can provide 4–5 orders of magnitude reduction in computing energy (Cassidy et al., 2014). To get further benefits, the neuromorphic core should be based on an analog resistive memory crossbar array. Both digital and analog neuromorphic cores will have an execution-time advantage as parallelism is easier to leverage in a neuromorphic computational model where communication latency is drastically decreased. Nevertheless, in this work we avoid focusing on a new parallel architecture and instead focus on demonstrating a more fundamental advantage in energy.

We will show that performing certain computations on an analog resistive memory crossbar provides fundamental energy scaling advantages over a digital memory based implementation for finite precision computations. This is true for any architecture that uses a conventional digital memory array, even a digital resistive memory crossbar. In addition we give a concrete neural-inspired application, sparse coding, which can be implemented entirely in analog and reap the aforementioned energy advantage. A rich neural-inspired problem is sparse coding (Olshausen, 1996; Lee et al., 2008; Arora et al., 2015), where one seeks to use an overcomplete basis set to represent data with a sparse code. It is used in many applications including computer vision, object tracking, and more generally unsupervised learning. We will show that analog neural-inspired architectures are ideally suited for algorithms like sparse coding, and outline an implementation of a specific sparse coding algorithm.

**Figure 1:**
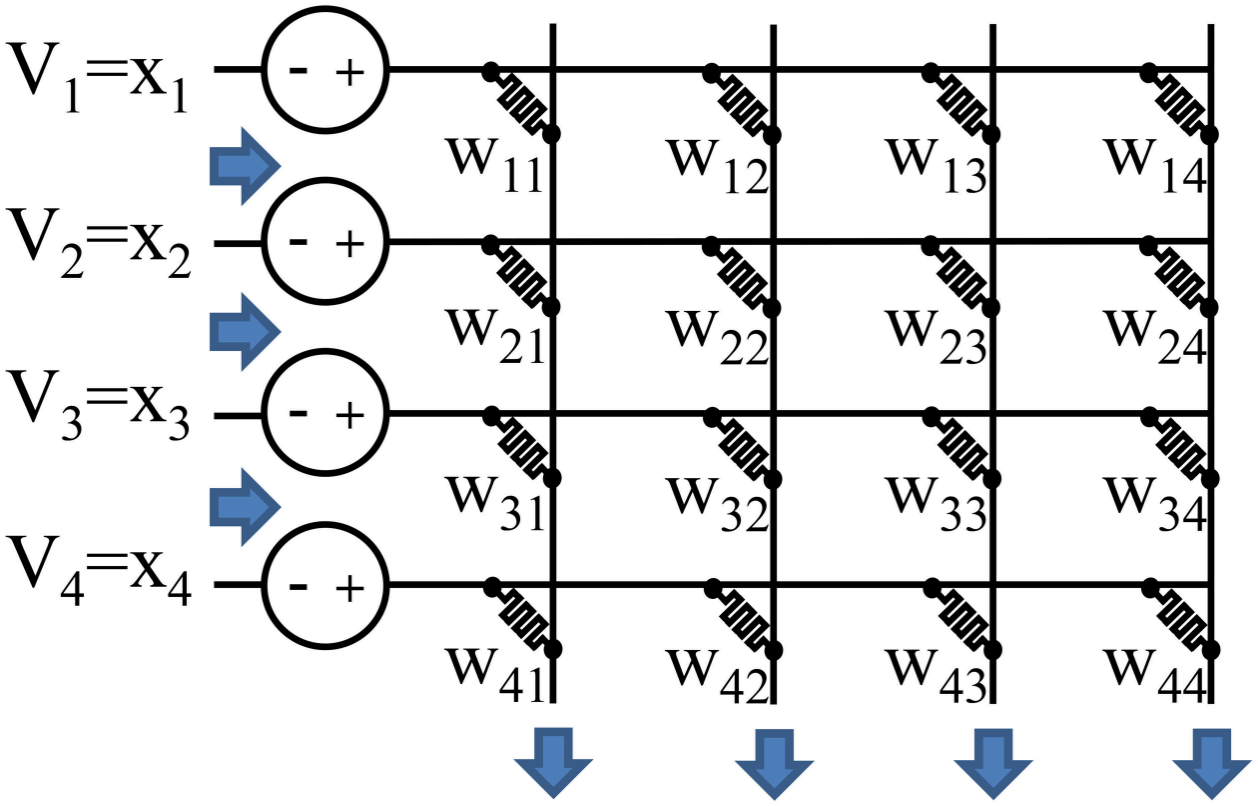
**Analog resistive memories can be used to reduce the energy of a vector-matrix multiply**. The conductance of each resistive memory represents a weight. Analog input values are represented by the input voltages or input pulse lengths, and outputs are represented by current values. This allows all the read operations, multiplication operations, and sum operations to occur in a single step. A conventional architecture must perform these operations sequentially for each weight resulting in a higher energy and delay.

Specifically, there are two key computational kernels that are more efficient on a crossbar. First, the crossbar can perform a parallel read or a vector-matrix multiplication as illustrated in Figure 1. Second, the crossbar can perform a parallel write or a rank-1 update where every weight is programmed based on the outer product of the row and column inputs. These two kernels form the basis of many neuromorphic algorithms.

In this paper we analyze the energy required to perform a parallel read and show that for a fixed finite precision, the noise limited energy to compute a vector dot product can be independent of the size of the vector, *O*(1), giving the analog resistive memory based dot product a significant scaling advantage over a digital approach. In the more likely situation of a capacitance limited energy, an *N* × *N* crossbar still has a factor of *N* scaling advantage over a digital memory. Similarly, writing a rank-1 update to a crossbar will also have a factor of *N* scaling advantage over a digital memory. We also analyze the energy cost of precision, energy scaling for communications, energy for accessing one row of the crossbar at a time and energy for accessing one element.

Next, we show that these computational kernels can be used with a sparse coding algorithm to make executing the algorithm *O*(*N*) times more energy efficient.

## Results

### Noise-limited parallel read

A resistive memory crossbar can be used to perform a parallel analog vector-matrix multiplication as illustrated in Figure 1. Each column of the crossbar performs a vector dot product: $\underset{i}{\sum }x_{i}w_{ij}$ for column *j*. The inputs, *x*_*i*_, are represented by either an analog voltage value or the length of a voltage pulse. The weights, *w*_*ij*_, are represented by the resistive memory conductances. The multiplication is performed by leveraging *I* = *G* × *V*, and the sum is performed by simply summing currents (or integrating the total current if the input, *x*_*i*_, is encoded in the length of a pulse).

The absolute minimum energy to read the crossbar will be determined by the thermal noise in each resistor. For many computations we only need to know the result with some finite precision. Taking advantage of this allows the minimum energy to compute the vector dot product to be independent of the size of the vector, *O*(1), when all the inputs and weights are positive.

To understand the tradeoff between precision and energy scaling, consider the minimum energy required to measure the current through *N* resistors with some signal to noise ratio (*SNR*). The signal strength we need to detect is dependent on the problem. If we want to keep the full precision of a digital computation, the minimum detectable signal must be proportional to the current through one resistor, *I*_*o*_. On the other hand, in many computations we only need to know the final result to some precision. The minimum detectable signal for positive inputs/weights will be proportional to *N* × *I*_*o*_. This means that we are throwing away extra information and no longer want to detect the change in a single input, *I*_*o*_. Effectively, we have a signal loss, α, of *N*, relative to a digital signal.

In many situations we will want negative weights or negative inputs. In this case the average signal might be zero. Nevertheless, the strength of the signal we want to detect will be given by the standard deviation of the signal. Consider inputs that have some distribution centered on zero, such as a Gaussian, and that have a variance proportional to $I_{o}^{2}$. The variance of *N* inputs will be proportional to *N* × $I_{o}^{2}$. The strength of the signal we are detecting will be given by $\sqrt{N}\times I_{o}$ and the loss relative to digital is $\sqrt{N}$. Overall, the signal strength we want to detect is α × *I*_*o*_ where α is between 1 and *N*.

The energy to read the resistors is given by:

(1) $$\begin{array}{l} \text{Energy}=\text{Power per resistor }\times \;N\text{ resistors }\times \text{time}\\ \;=V^{2}G_{o}\times N\times \frac{1}{\Delta f} \end{array}$$

*G*_*o*_ is the conductance of each resistor and *V* is the voltage used to read the resistors. The operation speed, Δ*f*, is determined by the thermal noise and the signal strength. We need to integrate the current for long enough to get the *SNR* we want. The thermal noise in *N* resistors is:

(2) $$\begin{array}{l} \text{Noise}=\langle \Delta I^{2}\rangle =N\times \left(4k_{b}T\times G_{o}\times \Delta f\right) \end{array}$$

The *SNR* is the signal strength divided by the noise:

(3) $$\begin{array}{l} SNR^{2}=\frac{{\left(\alpha I_{o}\right)}^{2}}{\langle \Delta I^{2}\rangle }=\frac{\alpha^{2}\times I_{o}^{2}}{4k_{b}T\times N\times G_{o}\times \Delta f} \end{array}$$

The current in a single resistor is given by *I*_*o*_ = *V* × *G*_*o*_. Using this and solving for time gives:

(4) $$\begin{array}{l} \frac{1}{\Delta f}=SNR^{2}\times \frac{4k_{b}T\times N\times G_{o}}{\alpha^{2}I_{o}^{2}}=SNR^{2}\times \frac{N}{\alpha^{2}}\frac{4k_{b}T}{V^{2}G_{o}} \end{array}$$

Plugging this back into Equation (1) gives:

(5) $$\begin{array}{l} \text{Energy}=V^{2}G_{o}\times N\times SNR^{2}\times \frac{N}{\alpha^{2}}\times \frac{4k_{b}T}{V^{2}G_{o}}\\ \;=4k_{b}T\times \frac{N^{2}}{\alpha^{2}}\times SNR^{2} \end{array}$$

For digital accuracy, α = 1, and the vector dot product energy is *O*(*N*^2^) and is *O*(*N*^3^) for the full crossbar.

For finite output precision with positive inputs/weights, α = *N* and so the vector dot product energy is *O*(1) and is *O*(*N*) for the full crossbar. *Thus, the total noise limited dot product energy is the same regardless of the crossbar size*. As we increase the number of resistors and therefore signal strength, we can measure each device faster and with less precision and energy per device to get the same precision on the output. This is summarized in Table 1.

**Table 1:** **Energy scaling for different precision requirements**.

	**Minimum detectable signal**	**Loss relative to digital (α)**	**Full crossbar noise limited read energy**
**Digital Accuracy**	*I*_*o*_	1	$N\times 4k_{b}T\times N^{2}\times SNR^{2}$
**Fixed Output Precision**	$\sqrt{N}\times I_{o}$	$\sqrt{N}$	$N\times 4k_{b}T\times N\times SNR^{2}$
**Fixed Output Precision, only positive inputs/weights**	*N* × *I*_*o*_	*N*	$N\times 4k_{b}T\times SNR^{2}$

### Capacitance-limited read

The previous analysis is only valid when the read energy is limited by the noise and not the capacitance. In particular, for fixed output precision with positive inputs/weights (α = *N*), this is when Equation (5) is greater than the energy to charge the resistive memory and wire capacitance:

(6) $$\begin{array}{l} 4k_{b}T\times SNR^{2}>N\times C_{\text{per}\;\text{RRAM}}V^{2} \end{array}$$

If we assume we have a 1000 × 1000 crossbar, want a *SNR* of 100, and a resistive memory dominated capacitance of 18 aF (20 × 20 nm area, 5 nm thick capacitor with a relative permittivity, ε_*r*_, of 25) we would need to perform the read at 100 mV or less to be noise limited. If a higher voltage is needed due to access devices or a larger crossbar is used, the energy will instead be capacitance limited.

For a capacitance-limited read energy, the crossbar will still be *O*(*N*) times more energy efficient than an SRAM memory. The scaling advantage occurs because in a conventional SRAM memory, each row or wordline must be read or written one at a time. This means that the columns/bitlines and associated circuitry will need to be charged *N* times for *N* rows. In an analog crossbar, everything can be done in parallel and so the columns/bitlines and associated circuitry are only charged once. Thus, the crossbar is *O*(*N*) times more energy efficient.

**Figure 2:**
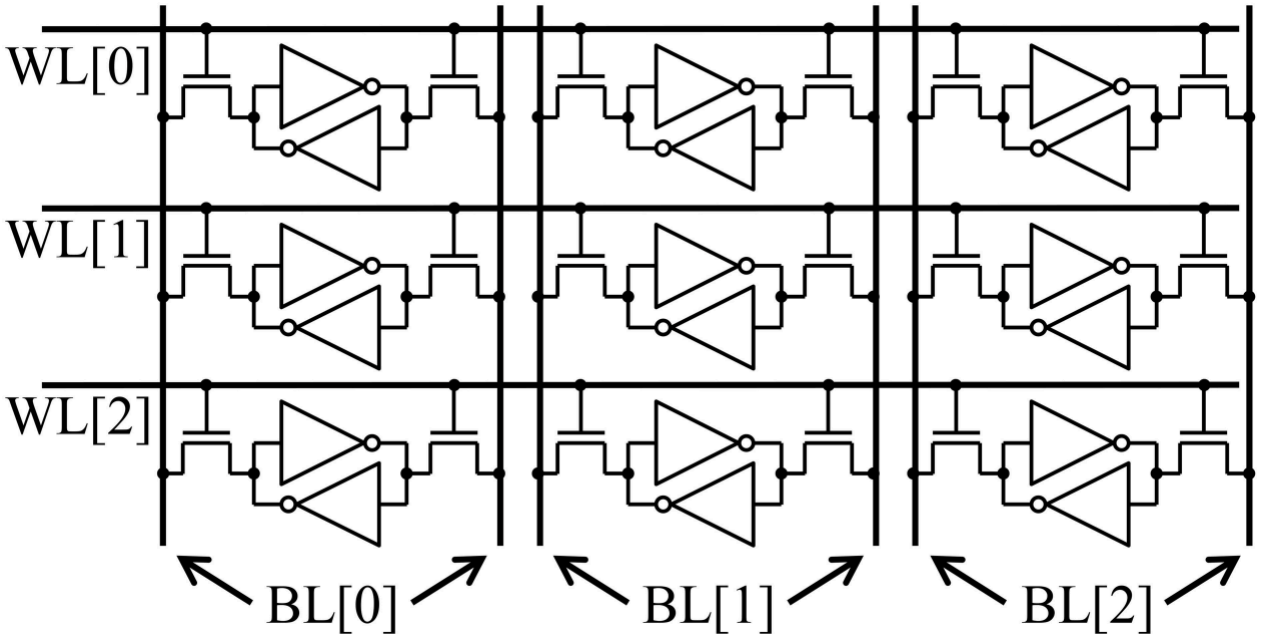
**A typical SRAM array**. Each row/wordline must be accessed sequentially.

The most energy efficient way to organize a digital memory for performing vector-matrix multiplication is to have the matrix stored in an SRAM (or even a digital resistive memory) array. The energy increases by orders of magnitude if the weights are stored off chip. A typical SRAM cache is illustrated in Figure 2. To perform a vector-matrix multiply, at best we can read out one row/wordline at a time. For an *N* × *N* array, there will be *N* memory cells along each row/wordline. To read each memory cell along a row, we need to charge each bitline/column and run the read electronics/sense amp for each cell. Thus, the total energy is:

(7) $$\begin{array}{l} \text{Energy }=\;N\text{ rows }\times N\text{ cells per row}\times E_{\text{digital bitline}}\\ \;=N^{2}\times E_{\text{digital bitline}}=O(N^{3}) \end{array}$$

The energy to charge each bitline, *E*_digital bitline_, is proportional to the capacitance and therefore the length of the bitline: $E_{\text{digital}\;\text{bitline}}=NC_{\text{cell}}V^{2}$ where *C*_cell_ is the line capacitance across a single resistive memory cell. Thus, the energy scales as *N*^3^.

In an analog resistive memory crossbar, all of the rows are charged in parallel and so the total energy is the sum of the energy to drive *N* rows and *N* columns:

(8) $$\begin{array}{l} \text{Energy }=\;N\text{ rows }\times E_{\text{analog row}}+N\text{ columns }\times E_{\text{analog column}}\\ \;=N\times (E_{\text{analog row}}+E_{\text{analog column}})=O(N^{2}) \end{array}$$

The energy to charge each line also scales as the length of each line and therefore as *N*. Thus, the total energy for a crossbar scales as *N*^2^ and is therefore is *O*(*N*) times more energy efficient than an SRAM memory.

When engineering memory systems, there are a number of tricks that can be used to try to engineer around the scaling limits. If on-chip optical communications become feasible, the entire scaling tradeoff will be far better as the communication energy will effectively become independent of energy. Unfortunately, the energy and area overhead in converting from electrical to optical is currently orders of magnitude too high (Miller, 2009). 3d stacked memories will also scale better. In that case, this analysis would apply to a single layer of a 3d stacked memory. Digital memories can be broken into smaller subarrays with a processing unit near each sub-array. This is the principle behind processing in memory architectures. Nevertheless, even a minimal multiplier and adder logic block takes up a significant amount of area, limiting the minimum memory array size required to amortize the logic cost. If logic blocks are not placed next to each subarray, the bus capacitance to each sub array will cause the same scaling limits. Adiabatic computing can be used to tradeoff speed for the capacitance limited energy for both the digital and analog approaches.

### Parallel write energy

The energy scaling to write a SRAM cell will be identical to the energy to read the cell, Equation (7). *N* rows must each be written one at a time, and each row has *N* cells. When writing each cell, the energy to charge the bitline will be proportional to *N*. Consequently, the energy to write the array will scale as *O*(*N*^3^).

**Figure 3:**
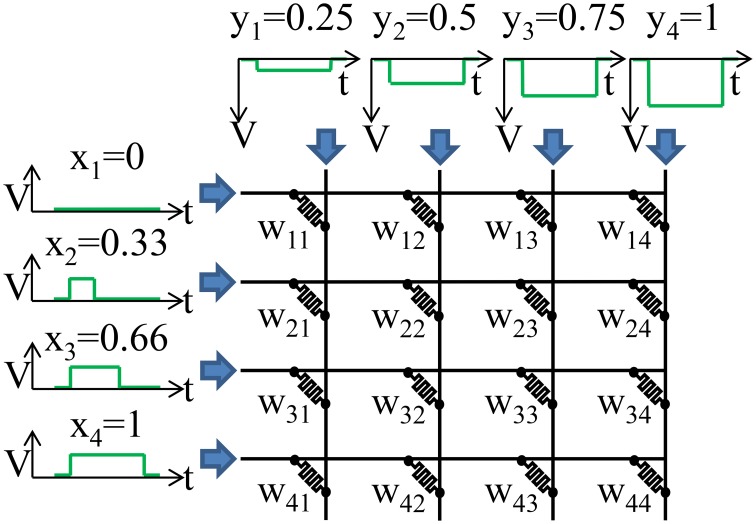
**A parallel write is illustrated**. Weight *W*_*ij*_ is updated by x_*i*_ × y_*j*_. In order to achieve a multiplicative effect the x_*i*_ are encoded in time while the y_*i*_ are encoded in the height of a voltage pulse. The resistive memory will only train when x_*i*_ is non-zero. The height of y_*i*_ determines the strength of training when x_*i*_ is non-zero.

On an analog crossbar, we can perform a “parallel write” or a rank-1 update where every weight is programmed based on the outer product of the row and column inputs. An example of a parallel write is illustrated in Figure 3. The goal is to adjust the weight, *W*_*ij*_, by the product of the inputs on the row, *x*_*i*_, and column, *y*_*j*_, of the weight:

(9) $$\begin{array}{l} W_{ij}^{\prime }=W_{ij}+x_{i}\times y_{j} \end{array}$$

An analog value for the row inputs, *x*_*i*_, can be encoded by the length of the pulse. The longer the pulse the more the weight will change. The analog column inputs, *y*_*j*_, can be encoded in the height of the pulse in order to achieve a multiplicative effect. The larger the voltage the more the weight will change for a given pulse duration. The exact write voltages will need to be adjusted to account for any non-linearities in the device. A parallel write can be done entirely in time as well (Kadetotad et al., 2015).

If the write is energy limited by the capacitance for the lines, the energy formula will be the same as in the read case and will be given by Equation (8). It will scale as *O*(*N*^2^) and is therefore is *O*(*N*) times more energy efficient than an SRAM memory. However, each resistive memory will also typically require a fixed amount of current to program. If the energy is limited by the program current, the total energy will be given by number of resistive memories times the energy to program one:

(10) $$\begin{array}{l} E_{\text{write}}=N^{2}I_{\text{write}}V_{\text{write}}\tau_{\text{write}} \end{array}$$

*I*_write_ and *V*_write_ are the current and voltage, respectively, required to write a resistive memory. τ_write_ is the time required to write the resistive memory. In this case the energy still scales as *O*(*N*^2^) and so it still is *O*(*N*) times more energy efficient than an SRAM memory.

If the write current or time is too large, it is possible that there will be a large constant factor that would make the energy scaling irrelevant. Fortunately, energies to fully write a resistive memory cell as low as 6 fJ have been demonstrated (Cheng et al., 2010). Furthermore, since we are operating the resistive memory as an analog memory with many levels, we do not want to fully write the cell. Rather, we only want to change the state by 1% or less, resulting in a corresponding reduction in the write energy per resistive memory. In this case the resistive memory energy will be on the same order of magnitude as the energy to charge the wires. (1% of 6 fJ is 60 aJ. The wire capacitance per resistive memory in a scaled technology node is likely to be on the order of 10's of attofarads [International Technology Roadmap for Semiconductors (ITRS, 2013)]. At 1V, that corresponds to 10's of attojoules as well).

### Energy cost of precision

So far we have ignored the energy cost of computing at high precision. Analog crossbars are best at low to moderate precision as seen below. There are three values that can each have a different level of precision. Let the inputs, *x*_*i*_, have a precisions in bits of *b*_in_, the outputs have a precision of *b*_out_, and the weights have a precision of *b*_*w*_. Consider the noise-limited parallel read energy. The energy per column is given by Equation (5) and is proportional to the *SNR*^2^ of the output. If we want $2^{b_{\text{out}}}$ levels on the output, the *SNR* must increase by $2^{b_{\text{out}}}$. This means that to create *N* outputs, at a precision of *b*_out_ bits, the crossbar energy will be on the order of the *O*(*N* $\times 2^{2b_{\text{out}}}$). If the crossbar is limited by the capacitance, the computation will already have sufficient precision and so the read/write energy will still be *O*(*N*^2^).

The thermal noise limited energy to process the output of the crossbar in analog at a certain precision will also scale as the voltage signal to noise ratio squared and therefore the number of output levels squared: $2^{2b_{\text{out}}}$(Enz and Vittoz, 1996). If the output is converted from analog to digital, the D/A energy typically scales as the number of levels, $2^{b_{\text{out}}}$ (Murmann and Boser, 2007). Similarly, to convert a digital input to analog will scale as the number of input levels: $2^{b_{\text{in}}}$. Thus, we see that in the capacitance limited regime the total energy to read the crossbar is on the order of:

(11) $$\begin{array}{l} \text{Analog Capacitance Limited Energy}=\text{O}\left(\text{N}\times \left(\text{N}+2^{2b_{\text{out}}}+2^{b_{\text{in}}}\right)\right) \end{array}$$

or in the noise limited regime with positive inputs and weights it is:

(12) $$\begin{array}{l} \text{Analog Noise Limited Energy}=O(N\times (2^{2b_{\text{out}}}+2^{2b_{\text{out}}}+2^{b_{\text{in}}}))\\ \;=O(N\times (2^{2b_{\text{out}}}+2^{b_{\text{in}}})) \end{array}$$

If we use a digital memory, we will need to store *b*_*w*_ bits for each weight. Consequently, we will need to multiply the energy by *b*_*w*_: *E* ~ *O*(*N*^3^*b*_*w*_). We will also need to multiply each weight by its input and then sum the result. Assuming *b*_*w*_ > *b*_in_, A single multiplication scales worse than *O*(*b*_*w*_ × log(*b*_*w*_)) (Fürer, 2009) and so an entire crossbar with *N*^2^ weights is at least *O*(*N*^2^ × *b*_*w*_ × log(*b*_*w*_)). The sum operation will scale slower. Assuming *b*_out_ < *b*_*w*_ × log(*b*_*w*_) any neuron operations will also scale slower than the multiply operations. Thus, the digital energy is:

(13) $$\begin{array}{l} \text{Digital Energy}=\text{O}\left(N^{2}\times b_{w}\times \left(N+\log\left(b_{w}\right)\right)\right) \end{array}$$

We see that for finite precision, analog is better, but if high floating point precision is required, digital is likely to be better.

### Communications energy

**Figure 4:**
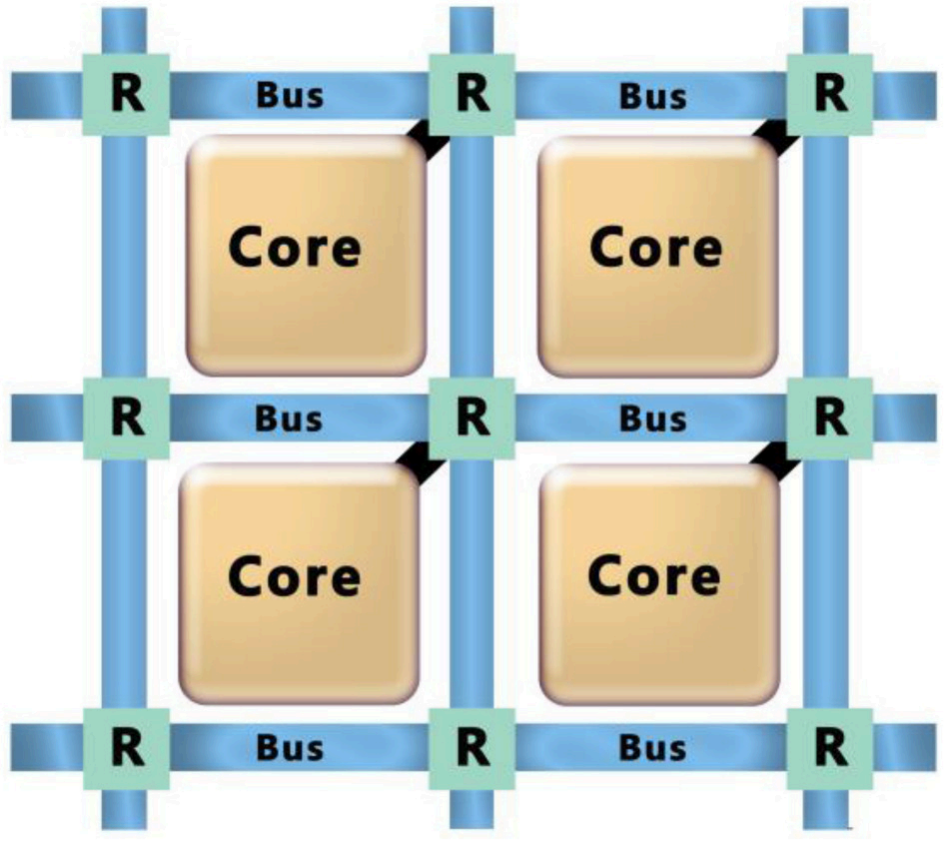
**A system would consist of individual crossbar based cores that communicate with each other through a communications bus and routing system**.

So far we have considered the energy of performing individual operations on a resistive memory crossbar. If we consider making a full system of multiple crossbars, the energy to communicate between crossbars can also be a significant component of the total energy. Consider the system shown in Figure 4. Each crossbar (or SRAM memory Merolla et al., 2014) is part of a neural core and each core communicates with the others over a communications bus. The energy to communicate between cores will be determined by the energy to charge the capacitance of the wire connecting two cores. Consequently the energy will be proportional to the capacitance and therefore the length of the wires. Assume that each core will communicate on average to a core that is fixed number of cores away. The size of each core will be determined by the size of the crossbar and so for an *N* × *N* crossbar, the length of an edge of a core will be of *O*(*N*). Similarly, the length of wire to go a fixed number of cores away is of *O*(*N*) and thus the energy is *O*(*N*).

The key kernels discussed so far assume that a single operation drives an *N* × *N* matrix with *N* inputs and has *N* outputs. That means that each operation will have *O*(*N*) communication events called spikes. Thus, we have *O*(*N*) spikes and *O*(*N*) energy cost per spike giving a total energy cost of *O*(*N*^2^). The energy to drive an SRAM based memory is of *O*(*N*^3^) and so the communications costs will be irrelevant for a large array. Indeed this is exactly the case in the IBM TrueNorth Architecture (Merolla et al., 2014). IBM projects that for an SRAM based system with a 256 × 256 core in a 10 nm technology the energy to communicate five cores away is 30 times lower than the energy to write the array. On the other hand, if we take advantage of an analog resistive memory crossbar, both the energy to read or write it and the energy to communicate will scale with *O*(*N*^2^). In this case, either the crossbar energy or the communication energy can dominate depending on the system architecture.

For algorithms that require cores that are far apart to communicate, the constant factor in the communication energy (the average communication distance) can be quite large and cause the communications energy to dominate. In this case, resistive memories can still provide a large constant factor reduction in the communication energy. Resistive memory potentially allows for terabytes of memory to be integrated onto a chip, while a chip using SRAM cannot hold more than 100 MB. This means that resistive memory can be >10,000*X* denser than SRAM. Consequently, the edge length of a core can be reduced by $\sqrt{10,000}=100$*X*. This would reduce the wire length and therefore communications energy by 100*X* or more. This is true regardless of whether the resistive memory is used as a digital or an analog memory.

### Sparse communications algorithms

So far we have only considered kernels that operate on the entire *N* × *N* core at once. Some algorithms only operate on 1 row or even 1 element at a time. In these cases the energy scaling is very different.

First, consider an algorithm that operates on a single row at a time. Assume that in a given step a core receives an input, reads and writes one row and then sends out one communication spike to another core. We assume that on average the number of input spikes is the same as the number of output spikes so that the system remains stable (the spikes don't die off over time or blow up so that everything is spiking all the time). In this case, both the digital and the analog energy to read/write the crossbar scale as *O*(*N*^2^). This is because *N* bitlines need to be charged for one row and the energy per bitline scales as *O*(*N*). Whether a digital or analog implementation is better will depend to the constant factors and exact system design. In both cases, using resistive memory for the memory reduces the wire lengths and therefore the power. The communications energy will scale as *O*(*N*) for a single spike in/out since the core edge length scales as *O*(*N*). This means the read/write energy will dominate as it scales as *O*(*N*^2^) and the communication scales as *O*(*N*).

Next, consider an algorithm that operates on only a single element. In a given step, a core receives an input, reads a single memory element and sends a single output. In both the analog and digital cases we will charge one bitline and one wordline and so the energy will be proportional to the length of the line and will be *O*(*N*). The communication energy for a single spike will also scale as *O*(*N*), proportional to the core edge length. Both the communications energy and core energy need to be simultaneously optimized as they both scale as *O*(*N*).

### Rectangular vs. square memory arrays

So far we have assumed all our memory arrays are square *N* × *N* arrays. Let's consider an *N* × *M* array with *N* rows and *M* columns. For an analog resistive memory, the capacitance-limited read/write energy will still scale as the length of each column times the number of columns, *O*(*N* × *M*).

For a digital memory each row must be accessed sequentially and so the energy will scale as the number of rows times the length of each column times the number of columns: *O*(*N*^2^ × *M*). If *N* << *M*, a digital rectangular array can be more efficient than a digital square array. Nevertheless, this is only true for a read if we only want output data along the *M* columns; i.e., we only perform the following multiplication, $\underset{i}{\sum }x_{i}w_{ij}$, where *i* represents the rows. If we also need to output data along the rows, i.e., perform the transpose operation: $\underset{j}{\sum }w_{ij}x_{j}$, the energy for that operation will scale as *O*(*N* × *M*^2^), which would be worse than a square matrix.

**Figure 5:**
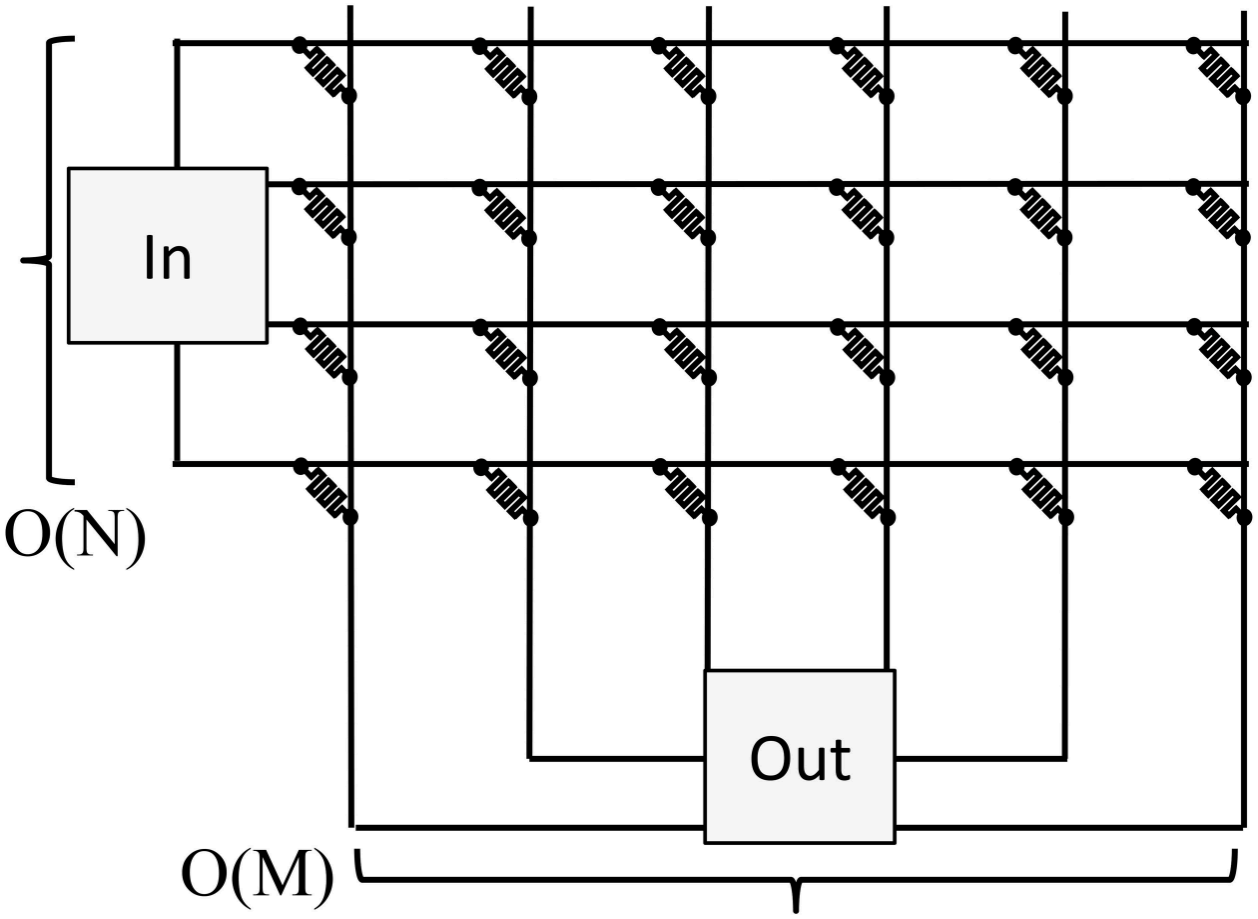
**If the data is not the same shape as the array, the input data will come from a single router, and the output data will need to go to a single computation unit**. At best, the extra wire length to go to the input/output units plus the row/column wire length will be *O*[max(*N*,*M*)].

In both cases, we have assumed that the data has the same shape, *N* × *M*, as the memory. This allows us to perform the sum operation at the edge of each array and minimize the data movement. If the data is not the same shape as the array, the energy will be worse. Consider the situation shown in Figure 5. When the data is not the same shape as the array, we will need to move the data to a computational unit at a single location. The average wire length going to that unit (including both the wires in the array and outside of it) will be *O*[max(*N*,*M*)]. Consequently, the energy will scale as the number of bits (*N* × M) times the total wire length *O*[max(*N*,*M*)] which is: *O*[(*N* × *M*) × max(*N*,*M*)]. In this case a square array with an edge length of sqrt(*N* × *M*) would be the most efficient with an efficiency of *O*(*N*^3∕2^ × *M*^3∕2^). The same energy scaling applies to a write operation: the value to be written to the array depends on both row and column inputs and so it must be computed in one location and then communicated to the bitlines/columns in the array.

### Sparse coding using a resistive memory array

The energy efficiency of a resistive memory array can directly translate to making an algorithm more energy efficient. Consider the problem of sparse coding. Sparse coding finds a set of basis vectors such that the linear combination of a few of these vectors is sufficient to explain each observation. Specifically, sparse coding finds matrix **A** that minimizes the following objective function (Olshausen, 1996; Lee et al., 2008):

(14) $$\sum_{k=1}^{p}{\left\|y_{k}-Ax_{k}\right\|}^{2}+\sum_{k=1}^{p}S(x_{k})$$

where **A** is an *M* by *N* matrix, *M* << *N*, of basis vectors, *p* is the number of observations, **y**_*k*_, (of size *M*) is observation *k*, **x**_*k*_ (of size *N*) is the sparse representation of **y**_*k*_, and *S* is a sparsity cost such as the *L*_1_ norm.

This problem is non-convex, but an approximate solution with guaranteed error bounds can be efficiently obtained via a recent algorithm by Arora et al. (2015) that extends the seminal gradient descent approach of Olshausen and Field (1997). In particular, we run *t* iterations or batches where we draw *p* samples and solve the following for each sample *k*:

(15) $$x_{k}=threshold_{C}\left(A{\left(t\right)}^{T}y_{k}\right)$$

where **A***(t)* is the sparse coding matrix at iteration *t*. *threshold*_*C*_*(.)* is a thresholding function that keeps coordinates whose magnitude is at least *C* and zeros out the rest ensuring the code **x** is sparse. Next we compute a matrix update, **Δg**_*k*_, which is the outer-product of two vectors:

(16) $$\Delta g_{k}=\left(y_{k}-A(t)x_{k}\right)\times \text{sgn}{\left(x_{k}\right)}^{T}$$

where sgn(.) is the sign function. All *p* updates need to be summed over a batch:

(17) $$g(t)=\sum_{k=1}^{p}\Delta g_{k}$$

At the end of each batch, *t*, we update the sparse coding matrix:

(18) $$A(t+1)=A(t)-\frac{\eta }{p}g(t)$$

where η is the learning rate.

This sparse coding algorithm can be implemented efficiently with two resistive memory arrays. One array stores the sparse coding matrix **A***(t)* while the second one stores the updates **Δg**_*k*_ during each batch. (Separate arrays should be used to minimize the wire length in each array) The arrays should be arranged to limit the wire length of the most frequent communication, *sgn(***x**_*k*_*)*, to be of *O*(*M*). This ensures that communications are not the limiting energy factor.

To analyze the energy efficiency, let's first consider all the operations performed for each sample *k*: For Equation (15), there are two operations being performed, the vector-matrix multiply, $\text{A}{\left(t\right)}^{T}{\text{y}}_{k}$, and the threshold function. In Equation (16), the resulting vector, **x**_*k*_, is multiplied by the matrix again, but without the transpose: **A**(*t*)**x**_*k*_. Then a vector subtraction is performed: **y**_*k*_ − **A**(*t*)**x**_*k*_. Next, a sign operation sgn(***x***_*k*_) is performed. Finally, we have two vectors that need to be multiplied in an outer product and added to second matrix that stores the weight update. Moving data to the second matrix will incur a communications cost. After p samples, the summed updates in the second matrix, **g***(t)*, need to be copied, multiplied by η/p and written back to the original matrix, **A***(t)*. This operation can only operate a single row at a time as each weight needs to be read, communicated and written independently. This means analog will not have benefit over digital for this operation. Fortunately, it is only performed once every p samples. All the operations and their energy scaling are summarized in Table 2. In analog all the matrix operations will cost *O*(*N* × *M*). To maximize the digital energy efficiency, we assume we arrange a digital memory to be a square giving and energy cost of *O*(*N*^3∕2^ × *M*^3∕2^).

**Table 2:** **The energy scaling for all the operations is given**.

**Operation**	**Analog energy scaling**	**Digital energy scaling**	**Repetitions per batch**
**MATRIX OPERATIONS**			
Multiplication: **A**(*t*)^*T*^ × **y**_*k*_	*O*(*N* × *M*)	*O*(*N*^3∕2^ ×*M*^3∕2^)	*p*
Multiplication: **A**(*t*) × **x**_*k*_	*O*(*N* × *M*)	*O*(*N*^3∕2^ ×*M*^3∕2^)	*p*
Multiplication/Training: $\left(y_{k}-A(t)x_{k}\right)\times \text{sgn}{\left(x_{k}\right)}^{T}$	*O*(*N* × *M*)	*O*(*N*^3∕2^ × *M*^3∕2^)	*p*
**VECTOR OPERATIONS**			
Threshold: *threshold*_*C*_ $\left(A{\left(t\right)}^{T}y_{k}\right)$	*O*(*N* × 2^*b*^)	*O*(*N* × *b*)	*p*
Subtraction: **y**_*k*_ − **A**(*t*)x_*k*_	*O*(*M* × 2^*b*^)	*O*(*M* × *b*)	*p*
Sign function: sgn(**x**_*k*_)	*O*(*N* × 2^*b*^)	*O*(*N* × *b*)	*P*
**COMMUNICATION**			
Vector: (**y**_*k*_ − **A**(*t*)x_*k*_)	*O*(*N* × *M*)	*O*(*N*^1∕2^ × *M*^3∕2^)	*P*
Vector: sgn(**x**_*k*_)^*T*^	*O*(*M* × *N*)	*O*(*N*^3∕2^ × *M*^1∕2^)	*P*
Matrix: **g**(*t*)	*O*(*N*^2^ × *M*)	*O*(*N*^3∕2^ × *M*^3∕2^)	1
**SERIAL OPERATIONS**			
Read: **g**(*t*)	*O*(*N*^2^ × *M*)	*O*(*N*^3∕2^ × *M*^3∕2^)	1
Write: $A(t+1)=A(t)-\frac{\eta }{p}g(t)$(*t*)	*O*(*N*^2^ × *M*)	*O*(*N*^3∕2^ × *M*^3∕2^)	1

*We consider the finite precision case such that 2^b^ < M*.

Let the inputs and outputs, have a precision in bits of *b*, and the weights have a precision of *b*_*w*_. We consider finite precision such that 2^*b*^ < *M*. This allows us to simplify Equation (11), the analog square matrix energy to be *E* ~ *O*(*N*^2^) and Equation (13) the digital energy to be *E* ~ *O*(*N*^3^). Here we are assuming that sparse coding algorithm will converge with a finite precision on the inputs and outputs. Neural-inspired algorithms like sparse coding tend to tolerate large amounts of noise, but the exact precision requirements should be studied for a practical implementation.

We can sum the energy scaling over all the operations listed in Table 2. Using the fact that 2^*b*^ < *M*<*N*<*p* (Arora et al., 2015) gives an overall analog energy scaling of: *O*(*N* × *M* × *p*) and an overall digital energy scaling of *O*(*N*^3∕2^ × *M*^3∕2^ × *p*). Thus, we see that analog has an overall energy advantage of *O*[(*N* × *M*)^1∕2^] or *O*(*N*) if *N* = *M*.

## Discussion

In this analysis we have deliberately avoided specifying constant factors as they can vary by orders of magnitude depending on the technology and design tradeoffs. Particular multiplicative constants apply only to today's hardware, but the big O remains whether new devices change these constants. For instance, the energy to write a resistive memory can be as low as 6 fJ (Cheng et al., 2010) or higher than 100 nJ (Mahalanabis et al., 2014). The energy for analog driving circuitry around a crossbar can also vary by orders of magnitude depending on the speed and circuit area tradeoffs. Depending on the algorithm, new semiconductor devices such as a spin based neuron (Sharad et al., 2014) could also drastically change the energy tradeoff.

Nevertheless, it is still useful to consider some specific numbers to understand what is plausible. In running an algorithm on a resistive memory array there are three key components to the energy, the parallel read energy, the parallel write energy and the energy for the driving circuitry. To find the capacitance limited read or write energy we need the capacitance per resistive memory element. The capacitance per element (wire + resistive memory) in an array for a 14 nm process as specified by ITRS will be around 50 aF. If we need to charge the wires to 1 V, that corresponds to 50 aJ per element. For an *N* × *N* array the total capacitance limited read or write energy would be 50 × *N*^2^ aJ. As discussed at the end of the Parallel Write Energy section, the current limited write energy could plausibly be on the same order of magnitude. The energy of the driving circuitry depends greatly on what computations are performed, but we can get an order of magnitude estimate by considering one of the most expensive analog operations, an analog to digital converter (ADC). ADCs that require only 0.85 fJ/level (or conversion step) have been demonstrated at 200 kHz (Tai et al., 2014). This means that for a 1000 × 1000 crossbar, the energy to run a six bit ADC is roughly the same as the energy to read/write a column of the crossbar. For higher precision ADCs, the ADC will dominate the energy, while for lower precision ADCs the crossbar will dominate the energy. In general, we see that the potential constant factors are on the same order of magnitude and consequently will be very technology dependent.

In order to understand the theoretical benefits of a crossbar, we have assumed ideal linear resistive memories. In practice there are many effects that can limit the performance of a resistive memory crossbar in a real algorithm. Access devices are required to be able to individually write a given resistive memory. This limits how low of a voltage can be used. Non-linearities in the resistive memories as well as those introduced by the access device mean that the amount a resistive memory writes will be dependent on its current state. Read and write noise limit the accuracy with which the resistive memories can be read or written. Parasitic voltage drops mean that devices far away from the drivers see a smaller voltage. Despite all of these effects, recent studies are indicating that iterative learning algorithms can tolerate and learn around moderate non-idealities (Burr et al., 2015; Chen et al., 2015; Cong et al., 2015). Given the potential energy scaling benefits of resistive memory crossbars, more work is need to design devices with fewer non-idealities and to better understand how various algorithms can perform given the non-idealities.

Overall, we have shown that the energy to perform a parallel read or parallel rank-1 write on an analog *N* × *N* resistive memory crossbar typically scales as *O*(*N*^2^) while a digital implementation scales as *O*(*N*^3^). Consequently, the analog crossbar has a scaling advantage of *O*(*N*) in energy. The communications energy between neighboring crossbars scales as *O*(*N*^2^). Thus, communications are not as important for digital approaches, but once we take advantage of an analog approach the communication energy and computation energy are equally important. For algorithms that operate on only one row of a matrix at a time, both the digital and analog energy scales as *O*(*N*^2^) per row. Therefore, the better approach will depend on the specifics of a given system. Algorithms such as sparse coding can directly take advantage of the parallel write and parallel read to get an *O*(*N*) energy savings.

Thus, we have shown that performing certain computations on an analog resistive memory crossbar provides fundamental energy scaling advantages over a conventional digital memory based implementation for low precision computations. This is true for any architecture that uses a conventional digital memory array, even a digital resistive memory crossbar. Fundamentally, a digital memory array must be accessed sequentially, one row at a time, while an entire analog memory crossbar can be accessed in parallel. Analog crossbars perform a multiply and accumulate at each crosspoint, while digital memories need to move the data to the edge of the array before it can be processed. In principle, a digital system could be organized to process data at every cell, but the area cost would become prohibitive. Alternatively, optimized digital neural systems will have a processing in memory (PIM; Gokhale et al., 1995) type architecture where simple operations are performed near a moderately sized memory array (Merolla et al., 2014). While this will give orders of magnitude reduction in energy compared to a CPU (Cassidy et al., 2014), the fundamental scaling advantages of an analog crossbar array can further reduce the energy by a few orders of magnitude.

## Author contributions

SA, TQ, OP, ED, MM, CJ, and JA designed research; SA, TQ, OP performed research; AH provided technical guidance; and SA, TQ, OP wrote the paper.

### Conflict of interest statement

The authors declare that the research was conducted in the absence of any commercial or financial relationships that could be construed as a potential conflict of interest.
